# CVID-associated intestinal disorders in the USIDNET registry: An analysis of disease manifestations, functional status, comorbidities, and treatment

**DOI:** 10.21203/rs.3.rs-2838051/v1

**Published:** 2023-05-08

**Authors:** Lauren E. Franzblau, Ramsay L. Fuleihan, Charlotte Cunningham-Rundles, Christian A. Wysocki

**Affiliations:** UT Southwestern: The University of Texas Southwestern Medical Center

**Keywords:** Common variable immunodeficiency, enteropathy, autoimmunity

## Abstract

Common variable immunodeficiency (CVID) has been subdivided into five phenotypes, including one marked by non-infectious enteropathies that lead to significant morbidity and mortality. We examined a large national registry of patients with CVID to better characterize this population and understand how the presence of enteropathy influences nutritional status, patient function, and the risk of additional non-infectious disorders in CVID patients. We also sought to illustrate the range of treatment strategies for CVID-associated enteropathies. We extracted patient data from the United States Immunodeficiency Network (USIDNET) database, which included 1415 patients with CVID, and compared those with and without intestinal disorders. Demographic and genetic profiles, functional status, and treatments targeting intestinal disorders are reported. Intestinal disorders were present in 20% of patients with CVID, including chronic diarrhea, inflammatory bowel disease, malabsorption, and others. Compared to those without enteropathies, this patient subset exhibited significantly lower Karnofsky-Lansky functional scores, greater reliance on nutritional support, higher rates of vitamin deficiencies, and increased prevalence of hematologic disorders, liver disease, pulmonary disease, granulomatous disease, and lymphoma. Genetic data were reported for only 5% of the cohort. No mutations segregated significantly to patients with or without intestinal disease. Corticosteroids were most frequently used for treatment. Patients with CVID-associated intestinal disorders exhibit higher rates of autoimmune and inflammatory comorbidities, lymphoma, malnutrition, and debility. We review recent studies implicating specific pathways underlying this immune dysregulation. Further studies are needed to evaluate the role of targeted immunomodulatory therapies for CVID-associated intestinal disorders.

## Introduction

Common variable immunodeficiency (CVID) is a primary immunodeficiency disorder marked by low immunoglobulin (Ig) levels and ineffective immune response to antigens leading to frequent infections. Many patients also suffer from autoimmune, inflammatory, and malignant complications driven by underlying immune dysregulation. This observation led to classification of CVID into five phenotypes, including autoimmunity, polyclonal lymphocytic infiltration, lymphoid malignancy, enteropathy, and no complications [1]. Enteropathies, which are non-infectious, inflammatory intestinal disorders, are thought to afflict 9–17% of patients with CVID [1–3]. They are linked to increased mortality [1, 4, 5]. Chronic diarrhea predominates and symptoms can mimic inflammatory bowel disease or celiac disease. In severe cases, malabsorption leads to dependence on parenteral nutrition [1, 4]. These conditions are notoriously challenging to treat and do not respond to immunoglobulin replacement [3, 6, 7]. Likewise, gluten withdrawal helps only 20% of patients and corticosteroids, while a mainstay of therapy, do not reliably lead to remission [3]. Recent case reports suggest that targeted biologic agents may be beneficial [3].

The histologic patterns in CVID-associated intestinal disorders are variable. One study identified a wide range of histologic patterns, mimicking lymphocytic colitis, collagenous enterocolitis, celiac disease, lymphocytic gastritis, granulomatous disease, acute graft-versus-host disease (GVHD), and inflammatory bowel disease [8]. Most studies examining this specifically have shown a predominance of villous blunting in the small intestine in 30–80% of patients, increased intraepithelial lymphocytes, mild duodenitis, and nodular lymphoid hyperplasia [7, 9–12]. Chronic gastritis is noted in up to 80%, with pernicious anemia reported [9, 11, 13–15]. In the colon, GVHD-like lesions are found with apoptosis and variably dense mononuclear cell infiltrates with obliteration of glands and crypts[9].

The pathophysiology of intestinal disease in CVID remains unclear. Mannon et al., found significantly higher interleukin (IL) 12 and interferon (IFN) gamma in stimulated lamina propria mononuclear cells from CVID patients with enteropathy [16]. Shulzhenko et al. also noted upregulation of type I and II IFN as well as a relative IgA deficiency in mucosa [17]. When compared to cells from patients with Crohn disease, similar levels of IL12 and IFN-gamma were produced, but significantly less IL23, IL17, and Tumor necrosis factor (TNF)a [16]. Thus, CVID-enteropathy involves a T helper (Th) 1 mediated inflammatory process driven by IL12 and IFN-gamma, which is distinct from Crohn disease. The role of dysregulated T cell mediated immunity is further substantiated by the prominence of severe enteropathy in CVID patients with cytotoxic T-lymphocyte associated protein 4 (CTLA4) insufficiency and LPS responsive beige-like anchor protein (LRBA) deficiency, known to cause abnormalities in regulatory T cell phenotype and function [18–26]. With this in mind a number of studies noted correlation between intestinal disease in CVID and numerous other autoimmune and inflammatory complications [9, 27–29].

CVID-related intestinal disorders appear to be linked to underlying immune dysregulation, yet the prevalence of inflammatory comorbidities is ill-defined. They remain challenging complications with detrimental prognostic implications. Here we report the prevalence and spectrum of non-infectious intestinal disorders among a large multicenter cohort of patients with CVID, collected through the United States Immunodeficiency Network (USIDNET). We also examine functional and nutritional status, genetic mutations, treatment strategies, and the prevalence of other inflammatory, autoimmune, and malignant comorbidities in this population. Finally we review the current understanding of immune dysregulation in CVID and implications for future therapies.

## Material and Methods

Data were obtained from the USIDNET database. This is a national repository of data from patients with primary immunodeficiencies which includes data entered by immunologists throughout the United States of America. Sixty-five independent contributors input 1417 patients with diagnoses of CVID. Of these 47.7% were input by the top three contributors, whereas 27 input only 1 patient each. Contributors input diagnostic, demographic, laboratory, and therapeutic data. However, because all fields are not mandatory some entries do not include all these data. We screened 1417 patients with CVID and excluded two based on genetic mutations suggesting alternative immunodeficiencies (Bruton tyrosine kinase and del22q11). The final cohort of 1415 patients was divided based on the presence or absence of non-infectious intestinal disorders that could be attributed to CVID. Infectious, acute, and nonspecific disorders were excluded. Supplementary Table S1 displays included intestinal disorders.

Functional and nutritional status were assessed with Karnofsky (age ≥ 16) or Lansky (age < 16) scores (these scales range from 0 [death] through 100 [full health]), body mass index, enteral or parenteral nutritional support, and nutritional deficiencies. We examined autoimmune, hematologic, pulmonary, hepatic, malignant, and granulomatous comorbid conditions. Autoimmune hematologic disorders were grouped by cell line, and malignancies were grouped by organ or cell line of origin. We also report the use of immunomodulatory medications among patients with intestinal disorders.

We compared demographic variables, CVID therapies, functional and nutritional status, and comorbid conditions between patients with and without intestinal disorders using Chi-square test for categorical variables and two tailed t-test for continuous variables. Comparisons in which there were fewer than 5 total patients between the groups being compared, and/or comparisons between 2 groups in which one group had 0 patients, were excluded from analyses. Significance was defined as p≤0.01 given multiple comparisons.

## Results

**Figure 1.**
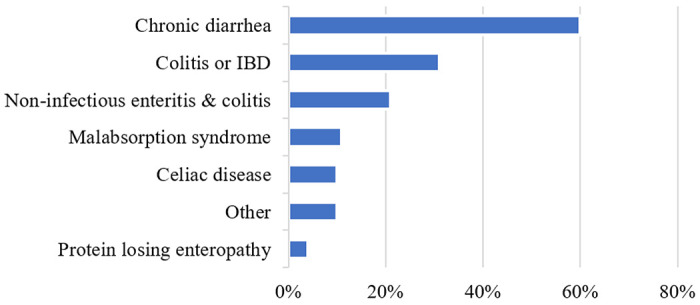
Distribution of Intestinal Disorders* *There is overlap between categories due to multiple diagnoses

Of 1415 patients with CVID, 290 (20%) had gastrointestinal disorders (GI cohort) and 1125 (80%) did not (non-GI cohort). The GI cohort carried a total of 431 diagnostic labels comprised of 21 unique intestinal disorders (Supplemental Table S1). Chronic diarrhea (60%) was most common and 31% had colitis or inflammatory bowel disease (Figure 1). In the GI cohort, 131 patients (45%) had documented upper and/or lower endoscopies. There were no differences in age, sex, or race between GI and non-GI cohorts, but those in the GI-cohort were more likely to be deceased (Table 1). Genetic mutations were reported for 5% (71 patients) (Supplemental Table S2). TNFRSF13B (TACI) mutations were the most common reported mutations but did not segregate statistically to either group. Among mutations found in more than one individual in the cohort, CTLA4 was the only gene mutated exclusively in patients in the GI cohort. There were no differences in mean serum IgA levels, mean IgM levels, or proportion of patients with decreased class switched memory B cells. Because 88% of patients received immunoglobulin therapy and baseline (pre-treatment) IgG levels were not known, IgG levels were not compared (Table 1).

### Nutritional and functional status:

There was no difference in body mass index between cohorts, though patients with intestinal disorders were more likely to receive enteral (p=0.001) or parenteral (p=0.007) nutritional supplementation (Table 2). The GI cohort also had a higher rate of vitamin D deficiency (p<0.001), and a trend toward more frequent vitamin B12 deficiency (p=0.07) (Table 2). No patients were noted as having copper deficiency. Karnofsky-Lansky scores were lower among the GI cohort compared to the non-GI cohort (80.2 vs 87.1, p=0.003).

### Comorbidities:

**Figure 2.**
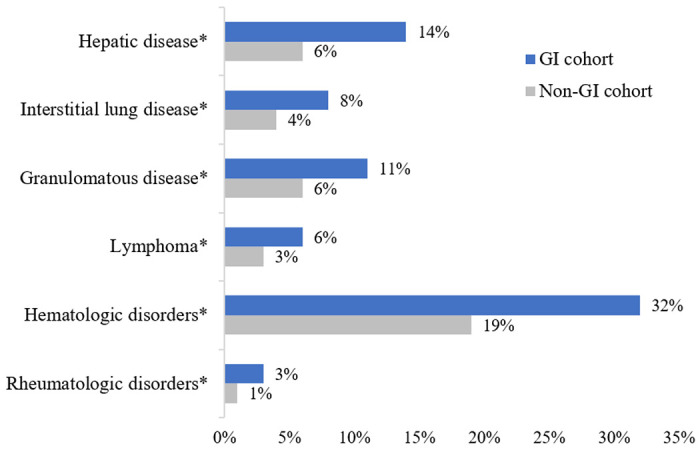
Comorbid conditions in GI and non-GI cohorts *p ≤0.01

Overall the GI cohort had more comorbidities, including hematologic disorders (p< 0.01), pernicious anemia (p<0.01), interstitial lung disease (p<0.01), lymphoma (p=0.01), liver disease (p<0.01), granulomatous disease (p<0.01), and rheumatologic disorders (p=0.01) (Figure 2). Detailed results are included in Supplemental Table S3. Hematologic disorders were significantly more prevalent in the GI cohort, including anemia and thrombocytopenia. Evans syndrome and neutropenia did not meet statistical significance. Interstitial lung disease was more prevalent in the GI cohort whereas other pulmonary diseases such as bronchiectasis and asthma were not. The GI cohort had higher rates of hepatic comorbidities, including autoimmune hepatitis, cirrhosis, granulomatous hepatitis, and liver failure. Rheumatologic diseases were increased overall in the GI cohort, with ankylosing spondylitis alone being significantly more prevalent. Lymphoma was significantly more prevalent in the GI cohort, while leukemia and solid organ malignancies were not. Granulomatous disease at any site was more prevalent among the GI cohort than the non-GI cohort (11% vs 6%, p<0.001).

### Treatment:

Immunomodulatory treatments were reported for 67 patients (23%) in the GI-cohort and 24 patients received multiple medications. Corticosteroids were the most common treatment followed by azathioprine, rituximab, infliximab, and mesalamine. Only 20 patients had data on the success of these interventions, with 18 reporting improvement and 2 no improvement (Table 3).

## Discussion

This large retrospective cohort analysis provides insights into the spectrum of non-infectious CVID-associated intestinal disorders and comorbid conditions. The overall prevalence of intestinal disorders was 20% with chronic diarrhea and colitis being the most common syndromes. Patients with intestinal disorders demonstrated poorer functional status, greater need for nutritional support, and higher prevalence of comorbidities, including lymphoma, autoimmunity, cytopenias, pulmonary, hepatic, and granulomatous disease. Interstitial lung disease, but not bronchiectasis was more common, supporting the notion that the autoimmune and inflammatory sequelae in these patients occur through mechanisms independent of recurrent infections. This study suggests a link between enteropathies and autoimmunity as well as lymphoproliferative disorders.

One in five patients in this study were diagnosed with intestinal disorders. This lies within prior cohorts based on tissue biopsy (3–15%) and patient-reported symptoms (47%) [1, 3, 7]. As in prior studies we found a spectrum of CVID related intestinal disorders including chronic diarrhea, inflammatory bowel disease, malabsorption, and protein-losing enteropathy. Compared to a retrospective chart review of 473 CVID patients, we found a higher prevalence of chronic diarrhea (60% vs 12%), a similar rate of inflammatory bowel disease (31% vs 27%), and a lower rate of malabsorption (11% vs 38%) [4]. This variability may be in part due to methodological differences in defining intestinal disorders, which lack standardized diagnostic criteria. Patients with CVID-associated intestinal disease had lower Karnofsky-Lansky scores, indicating greater morbidity and disability. This is likely a multifactorial result of enteropathy, malnutrition, and frequent autoimmune, inflammatory, and malignant comorbidities. Prior studies have also observed overlap between CVID-associated intestinal disease and other autoimmune and inflammatory disorders, which suggests underlying immune dysregulation in this subset of CVID patients [1, 9, 27–29].

Multiple potential pathways have been proposed to cause immune dysregulation in CVID patients. CD21^low^ B cells are polyclonal IgM + IgD + B cells which produce autoreactive IgM antibody, and are shown to possess unique properties including the capability of homing to peripheral tissues such as synovium or lungs [30]. These cells are found to be elevated in patients with autoimmune disorders such as rheumatoid arthritis and lupus, as well as in CVID patients with autoimmunity and lymphoproliferation (data on this cell population were not available for analysis in our cohort) [30, 31].

It has also been proposed that decreased diversity of gut microbiota and impaired intestinal barrier function in CVID patients may lead to microbial translocation and chronic immune activation. CVID patients have higher serum levels of bacterial lipopolysaccharide, soluble CD14, and IL2 supporting increased translocation [32]. The altered microbiota has been attributed in part to low local IgA in the intestinal mucosa in CVID patients with enteropathy, although interestingly (as in this study), total serum IgA levels are similar in CVID patients with or without intestinal disease [17].

T cells in CVID patients with inflammatory disorders exhibit a Th1 skewed phenotype, increased markers of activation and proliferation, as well as reduced CD4/CD8 ratio, reduced naïve T cells, and impaired regulatory T cell (Treg) function [31, 33]. Th1 cytokines and chemokines (CXCL9, CXCL10, CXCL11)are increased, Th17 cytokines are increased, chemokines and receptors selective for mucosal sites (CCL20, CCR9) and lymphoid tissue (CCL19) are increased, as well as integrins, and monocyte chemotactants (Receptor activator of nuclear factor kappa B ligand, or RANK-L), which may facilitate antigen independent migration, tissue infiltration, and end organ damage [33]. Immune regulatory markers are also increased including IL10, lymphocyte activation gene-3 (LAG3), TNFRSF9, and CD83, suggesting chronic immune activation. Interestingly, terminally differentiated T cells retained expression of IFN gamma, Ki67, and/or Granzyme B, indicating continued functional capacity and not exhaustion [33]. While some studies have found lower Treg numbers in CVID, others have noted preserved Tregs with functional failure marked by lack of CTLA4 upregulation [20, 31, 33]. These studies suggest that lack of T cell regulation coupled with sustained Th1 activation along with local and systemic autoantibody production may combine to cause the multiorgan autoimmune and inflammatory phenotype we are seeing in patients with CVID and intestinal disease. It is possible that this process could be initiated and/or propagated through impaired intestinal barrier function, providing a “link” between intestinal disease and other autoimmune and inflammatory disorders in these patients.

The results of our study highlight the need for close follow-up and screening of CVID patients with intestinal disease. Based on this clustering of comorbidities, patients with intestinal disorders should be screened and followed for nutritional deficiencies, hematologic disorders, pulmonary and hepatic dysfunction, and osteopenia/osteoporosis [34, 35].

Based on this understanding of immune dysregulation and genotyping of CVID patients, treatment of inflammatory complications is shifting from corticosteroids to more targeted immunomodulation. While we found that corticosteroids were used more frequently than targeted biologics or other immunosuppressive agents, this cohort does not offer conclusive data regarding the efficacy of these interventions. There is some evidence to suggest that CVID patients with mutations in immunoregulatory genes, such as CTLA4, are more prone to autoimmune and inflammatory complications including intestinal disorders [18, 19, 24]. In patients with autoimmune features, targeted or whole exome sequencing to identify these mutations could help to guide therapy. For example, one patient in this cohort with a CTLA4 mutation had improvement in GI symptoms with abatacept, echoing recent case series and reports of CTLA4 haploinsufficient patients who received abatacept or belatacept [24, 36, 37]. Similar targeted therapy has been reported in patients with signal transducer and activator of transcription (STAT)-3 gain of function in which tocilizumab (anti-IL6 monoclonal antibody) with and without Janus kinase inhibitor was shown to control inflammatory complications.[38, 39] Additionally, the known Th1 pathway involvement in CVID related enteropathy suggest that targeting IFN-gamma and IL12 with either monoclonal antibodies or downstream inhibitors such as Janus kinase inhibitors could be beneficial [16].

This study has several limitations. Data in the USIDNET database are input at the discretion of provider-contributors and not systematically collected. The diagnostic labels in our cohort come from individual clinicians and may be based on clinical, laboratory, and/or endoscopic findings. It is unclear how many patients were endoscopically evaluated. Some data were not available for all patients. For example, treatment modalities and genetic mutations were reported for less than one third of patients. Where we did have genetic data, we compared results to the diagnostic label and excluded patients with incompatible results (e.g. Bruton tyrosine kinase mutation which is incompatible with CVID or positive testing for infectious enteropathies such as rotavirus). Nutritional deficiencies are likely underreported as not all patients were tested for these. Nonetheless this is one of the largest published cohorts of patients with CVID and associated intestinal disorders.

## Conclusions

In this large national cohort of patients with CVID, 20% had enteropathies. This subset of patients demonstrates a high burden of autoimmune and inflammatory disorders as well as lymphoma. They also exhibit lower functional status and greater reliance on nutritional support. Ideal therapeutic approaches remain elusive. As our understanding of the immune dysregulation affecting this subset of CVID patients grows, new strategies based on genetic, proteomic, and cytokine signatures have come to light. Further studies are needed to evaluate these treatment options particularly among steroid refractory enteropathies which contribute to morbidity and mortality. Because of the rare nature of CVID-related intestinal disorders, national data repositories like USIDNET and multi-site collaborations will be critical to gathering sufficient numbers of patients to study the efficacy of these treatments.

## Figures and Tables

**Table 1.** Demographic characteristics

	Entire Cohort	GI Cohort	Non-GI Cohort	
	N = 1415	N=290	N=1125	
	No. (%)	No. (%)	No. (%)	p value
*Male*	544 (38%)	121 (42%)	423 (38%)	
*Female*	871 (62%)	169 (58%)	702 (62%)	
*Age or age at death (mean, yrs.)*	45.3	44.2	45.6	
*Deceased* *	46 (3%)	17 (6%)	29 (3%)	0.006
*Race*				0.424
*White*	1116 (79%)	232 (80%)	884 (79%)	
*Black*	23 (2%)	7 (2%)	16 (1%)	
*Asian*	7 (0.5%)	3 (1%)	4 (0.4%)	
*Other*	28 (2%)	5 (2%)	23 (2%)	
*Unknown*	241 (17%)	43 (15%)	198 (18%)	
*Hispanic ethnicity*	35 (3%)	8 (3%)	27 (2%)	
*Immunoglobulin therapy* *				0.009
*Yes*	1245 (88%)	277 (96%)	968 (86%)	
*No*	69 (5%)	7 (2%)	62 (6%)	
*Unknown*	101 (7%)	6 (2%)	95 (8%)	
*HSCT*	21 (1%)	3 (1%)	18 (2%)	
*IgA levels (mean, mg/dl)*	68	67	68	0.939
*IgM levels (mean, mg/dl)*	75	81	73	0.610
*Class switched memory B cells*				0.906
*<2%*	94 (39%)	20 (38%)	74 (39%)	0.927
*≥2%*	146 (61%)	32 (62%)	114 (61%)	0.941

BMI = body mass index, HCST = hematopoietic stem cell transplant, Ig = immunoglobulin

* p≤0.01

**Table 2.** Nutritional and Functional Status

	Entire Cohort	GI Cohort	Non-GI Cohort	
	N = 1415	N=290	N=1125	
	No. (%)	No. (%)	No. (%)	p value
*Karnofsky-Lansky Score (mean)* *	85.4	80.2	87.1	0.003
*Nutritional Status*				
*BMI (mean, kg/m^2^)*	26.7	26.5	26.7	0.845
*Enteral supplements* *	23 (2%)	11 (4%)	12 (1%)	0.001
*Parenteral nutrition* *	17 (1%)	8 (3%)	9 (1%)	0.007
*Vitamin B12 deficiency*	6 (0.4%)	3 (1%)	3 (0.3%)	0.07
*Vitamin D deficiency* *	33 (2%)	20 (7%)	13 (1%)	<0.001

* p≤0.01

**Table 3.** Treatment of CVID-associated intestinal disorders with improvement status after treatment

Medication	No. Patients*	No. Improved	No. Not improved
Corticosteroid	53	7	1
Azathioprine	7	2	
Rituximab	7		
Infliximab	6		1
Mesalamine	6	3	
Sirolimus	5		
Hydroxychloroquine	3		
Mercaptopurine	3		
Methotrexate	3	1	
Abatacept	2	1	
Mycophenolate	2	1	
Sulfasalazine	2		
Tacrolimus	2	1	
Adalimumab	1	1	
Cyclosporine	1	1	
Sertraline	1		
Vedolizumab	1		

* Treatments modalities were reported for 67 of 290 patients in the GI-cohort. Improvement status was reported for 17 of these patients.

## Data Availability

This manuscript data is not deposited in a data repository.
